# Solution NMR of MPS-1 Reveals a Random Coil Cytosolic Domain Structure

**DOI:** 10.1371/journal.pone.0111035

**Published:** 2014-10-27

**Authors:** Pan Li, Pan Shi, Chaohua Lai, Juan Li, Yuanyuan Zheng, Ying Xiong, Longhua Zhang, Changlin Tian

**Affiliations:** 1 Hefei National Laboratory of Microscale Physical Sciences, School of Life Sciences, University of Science and Technology of China, Hefei, Anhui, P. R. China; 2 High Magnetic Field Laboratory, Hefei institutes of Physical Science, Chinese Academy of Sciences, Hefei, Anhui, P. R. China; George Washington University, United States of America

## Abstract

*Caenorhabditis elegans* MPS1 is a single transmembrane helical auxiliary subunit that co-localizes with the voltage-gated potassium channel KVS1 in the nematode nervous system. MPS-1 shares high homology with KCNE (potassium voltage-gated channel subfamily E member) auxiliary subunits, and its cytosolic domain was reported to have a serine/threonine kinase activity that modulates KVS1 channel function via phosphorylation. In this study, NMR spectroscopy indicated that the full length and truncated MPS-1 cytosolic domain (134–256) in the presence or absence of n-dodecylphosphocholine detergent micelles adopted a highly flexible random coil secondary structure. In contrast, protein kinases usually adopt a stable folded conformation in order to implement substrate recognition and phosphoryl transfer. The highly flexible random coil secondary structure suggests that MPS-1 in the free state is unstructured but may require a substrate or binding partner to adopt stable structure required for serine/threonine kinase activity.

## Introduction

Voltage-gated potassium (Kv) channels are widely expressed in nervous, cardiovascular and other tissues, and are crucial mediators of membrane excitability in virtually all mammals [1], [2]. The highly diverse functions of Kv channels originate partly from the pore-forming α subunits. Additional regulatory proteins or β-subunits also modulate Kv channel properties including expression and distribution, sensitivity to stimulation, gating and pharmacological responses [3], [4]. MinK-related peptides (MiRPs) are well-characterized KCNE family membrane proteins associated with modulation of voltage-gated potassium channels in heterologous systems such as the HERG, KCNQ1, HCN, and Kv4.2 subunits [5]–[8]. MPS-1 belongs to the conserved KCNE family of β-subunits and is the first MiRP-related β-subunit to be investigated in the *nematode Caenorhabditis elegans*. Four MPS-1, MPS-2, MPS-3 and MPS-4 are expressed exclusively in the *C. elegans* nervous system [9] and are essential for neuronal excitability [10].

MPS-1 is a single transmembrane domain β-subunit that modulates the voltage-gated pore-forming potassium channel KVS1 in *C. elegans* [11]. Secondary structure prediction indicates a typical mammalian MiRP-like topology consisting of an extracellular N-terminus, a single transmembrane domain, and an intracellular C-terminus [10]. MPS-1 colocalizes with KVS-1, and this complex was shown to conduct less current, exhibited faster inactivation, and recovered slower from inactivation when compared to the KVS-1 alone [9]. Defects in either MPS-1 or KVS-1 can result in defective chemotaxis, disrupted mechano-transduction, and impaired locomotion [10].

As with other KCNE β-subunit family, the transmembrane domain of MPS-1 is both necessary and sufficient for MPS-1-KVS-1 complex formation [12]. Unlike other KCNE β-subunits, the cytoplasmic domain of MPS-1 was reported to possess serine/threonine kinase activity, and phosphorylation of KVS-1 by MPS-1 lowered the ability of the KVS-1 channel to be opened [13]. Sequence homology with AGC protein kinases (a family of protein kinase containing PKA, PKC and PKG)[14] shows two characteristic protein kinase motifs in the cytoplasmic domain: (1) the DFG (Asp-Phe-Gly) triplet, which contributes to the Mg^2+^ binding site of ATP-binding molecules, and (2) the HSD (His-Ser-Asp) triplet, which contributes to the catalytic function [14], [15]. Mutating D178 to N in the Δ132-256 truncated version of MPS-1 abolished KVS-1 channel modulation [13], suggesting the MPS-1 cytosolic domain is essential for KVS-1 phosphorylation and consequent channel modulation. Auto-phosphorylation has also been reported for MPS-1, similar to observation with other protein kinases [16]. Consequently, two independent KVS-1 channel modulation mechanisms have been proposed to explain how MPS-1 may reduce the channel current. The first mechanism involves formation of a complex between KVS-1 and the MPS-1 transmembrane helices, as observed with other KCNE β-subunits. The second mechanism involves phosphorylation of KVS-1 by the serine/threonine kinase activity of the MPS-1 cytosolic domain [13]. Results of the current studies have provided insights in the latter mechanism.

In addition, MPS-1 was also reported to assemble with the voltage-gated K^+^ channel KHT-1 (K^+^ channel for habituation to tap) in the tactile neurons of nematodes. MPS-1 was reported to phosphorylate KHT-1 and modulation of KHT-1 current channeling properties was observed [17].

**Figure 1:**
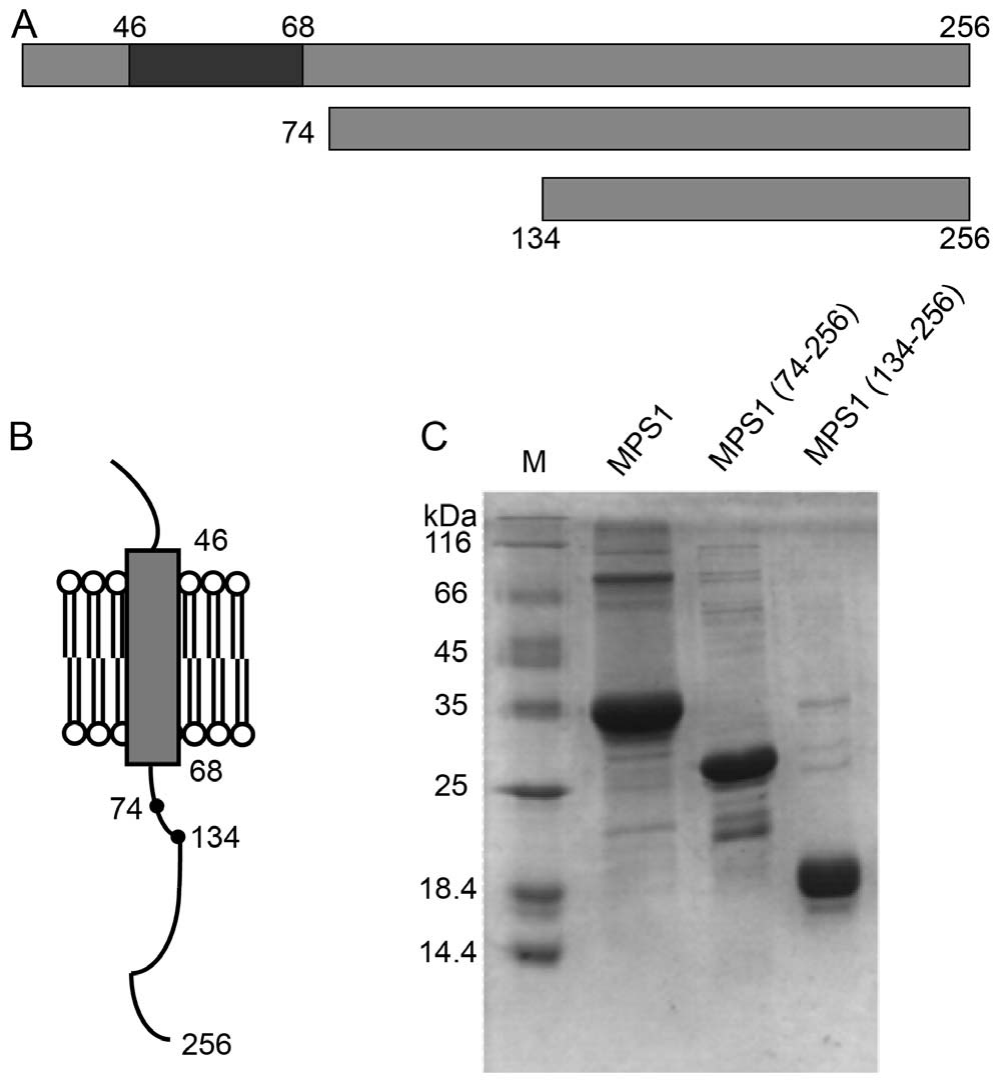
Schematic diagram of full length and truncated MPS-1. (A) Various MPS-1 constructs with MPS-1(1–256), MPS-1(74–256) and MPS-1(134–256) depicted from top to bottom. The black segment (residues 46 to 68) represents the predicted transmembrane helix; (B) Model of the MPS1 domains showing the two phosphorylation sites within the cytoplasmic C-terminal domain. (C) SDS-PAGE of the purified MPS-1 proteins. Lane 1: molecular weight marker; lanes 2-4: purified MPS-1(1–256), MPS-1(74–256) and MPS-1(134–256).

Despite numerous electrophysiological and biochemical studies on the interactions between MPS-1 and KVS-1 or other Kv channels, no structural studies have yet been performed that can probe the MPS-1 channel modulation mechanism at the atomic level. To this end, the structural properties of full length MPS-1(1–256) and two truncated versions (74–256; 134–256) were studied by NMR spectroscopy (Fig. 1A, B). The MPS-1(74–256) variant included an almost intact cytosolic domain, whereas the MPS-1(134–256) variant, prepared based on previous observations, abolished the KVS-1 channel modulation activity exhibited by the Δ132–256 MPS-1 variant [13]. Our studies included the use of n-Dodecylphosphocholine (DPC) detergent micelles to mimic the lipid bilayer environment of the cell membrane, since the choline head group of DPC is similar to those of native lipids. Surprisingly, secondary structure and backbone relaxation analysis indicated that the cytosolic domain adopted a highly flexible random coil secondary structure that is inconsistent with the stable fold required of a functional serine/threonine kinase. These results question the phosphorylation-based channel modulation mechanism, and suggest that further *in vivo* biophysical and protein–protein interaction experiments are required.

## Materials and Methods

### Cloning, Over-expression and Purification of Full-length and Truncated MPS-1

A DNA fragment encoding full-length MPS-1 was amplified by PCR from a *C. elegans* cDNA library. Truncation fragments MPS-1(74-256) and MPS-1(134–256) were amplified using pairs of primers with *Nde* I and *Xho* I at the 5′- and 3′-ends, respectively. The amplified full-length and truncated fragments were ligated into the pET-21b vector (Novagen, Co.). All constructs were confirmed by DNA sequencing. Expression constructs were transformed into BL21(DE3) Rosetta (Novagen Co.) and cells were incubated overnight at 37°C on Luria-Bertani (LB) agar containing ampicillin and chloramphenicol. Colonies were transferred to 4 mL LB medium containing ampicillin and chloramphenicol and incubated at 37°C overnight with shaking at 225 rpm. This culture was used to inoculate M9 minimal medium containing 100 µg/ml ampicillin and 100 µg/ml chloramphenicol, and cultures were grown at 37°C, 225 rpm until the OD_600_ reached 0.6. Flasks were then incubated at 25°C until the OD_600_ reached 0.8, and IPTG was added to a final concentration of 0.8 mM to induce protein expression, and the culture was incubated at 25°C for 12 h.

To achieve uniform isotope labeling during protein expression, 1 g/L [^15^N]-NH_4_Cl and 3 g/L [^13^C] labeled glucose (Cambridge Isotope Laboratory, Andover, MA, USA) were added to M9 medium for ^13^C/^15^N labeling. For ^15^N labeling only, unlabeled glucose was used.

Full length MPS-1(1–256) was expressed as inclusion bodies and bacterial cells were harvested by centrifugation at 4000 rpm for 20 min at 18°C. Cell pellets were resuspended in 40 mL lysis buffer (70 mM Tris–HCl, 300 mM NaCl, pH 8.0) and sonicated on ice using a VC500 probe sonicator (Sonics and Materials, Danbury, CT, USA) for a total of 10 min at a power level of 30% using 2 s pulses with 4 s between pulses. Five milligrams lysozyme, 1 mg DNase and 0.5 mg RNase were added, and the lysate was mixed at 4°C for 1 h before centrifugation at 16,000 rpm for 20 min at 4°C. The Pellet was washed three times by resuspending in 40 mL lysis buffer and centrifuged. The pellet was then resuspended in binding buffer (20 mM Tris–HCl, 100 mM NaCl, pH 8.0) containing 8 M urea and 0.2% (w/v) SDS and incubated at room temperature (25°C) for 2 h followed by centrifugation at 16,000 rpm for 20 min at 18°C to remove any insoluble debris. The supernatant was mixed with 5 mL Ni^2+^-NTA resin (QIAgen, Valencia, CA, USA) and loaded onto a gravity-flow column (BIO-RAD, Hercules, CA, USA) equilibrated in 20 mM Tris–HCl, 200 mM NaCl, pH 8.0. Weakly bound *E. coli* proteins were eluted using 50 mL 20 mM Tris–HCl, 200 mM NaCl, 8 M urea, 0.2% (w/v) SDS, pH 8.0, followed by 40 mL wash buffer (20 mM Tris–HCl, 200 mM NaCl, pH 8.0) containing 0.2% (v/v) DPC (Anatrace, Maumee, OH, USA). On-column refolding of the protein was achieved during this step while the detergent was exchanged from SDS to DPC. Full-length MPS-1 was eluted using elution buffer (20 mM Tris–HCl, 200 mM NaCl, 250 mM imidazole, pH 8.0, 0.5% DPC).

The MPS-1(74–256) and MPS-1(134–256) truncations were over-expressed as soluble proteins in *E. coli*. Cells were harvested by centrifugation, suspended in lysis buffer, and lysed by sonication as described above. After centrifugation, supernatants were mixed with 5 mL Ni^2+^-NTA resin (QIAgen) at 4°C for 30 min before loading onto a gravity-flow column (BIO-RAD) equilibrated with 20 mM Tris–HCl pH 8.0, 200 mM NaCl. Weakly bound *E. coli* proteins were eluted with 50 mL 20 mM Tris–HCl, 200 mM NaCl, pH 8.0, then with 40 mL wash buffer (20 mM Tris–HCl, 200 mM NaCl, 30mM imidazole, pH 8.0). The truncated proteins were eluted with 20 mM Tris–HCl, 200 mM NaCl, 250 mM imidazole, pH 8.0.

Protein concentration was determined by measuring the absorbance at 280 nm, and the purity was checked using standard SDS-PAGE. Purified proteins were concentrated using an Amicon Ultra-15 device with a 3000 MWCO (Millipore), and this was also used for buffer exchange into NMR buffer (50 mM NaH_2_PO_4_/Na_2_HPO_4_, pH 6.5). The final sample volume was 450 µL, to which 50 µL D_2_O was added to yield a final concentration of 10% (v/v). A protein concentration of 1.0 mM was used for the multi-dimensional NMR experiments. 100 mM DPC was added to an equivalent [^13^C, ^15^N] labeled MPS-1(134–256) sample for multi-dimensional NMR experiments.

### Solution NMR Data Acquisition and Backbone Resonance Assignment

Two-dimensional ^1^H-^15^N hetero-nuclear single quantum correlation spectroscopy (HSQC) experiments on ^15^N-labeled full-length and truncated protein samples was performed at 25°C in a 600 MHz Bruker spectrometer equipped with a TXI probe. Standard Bruker HSQC pulse sequences with careful 90° pulse calibration were applied for data acquisition with 1024×256 complex points in the ^1^H and ^15^N dimensions, respectively. The acquired HSQC data were processed using nmrPipe [18] with a Gaussian window function in both dimensions and spectra were analyzed using nmrView [19].

A set of triple resonance multi-dimensional NMR experiments were conducted at 25°C for the [^13^C, ^15^N] labeled MPS-1(134–256) sample in 50 mM NaH_2_PO_4_/Na_2_HPO_4_, pH 6.5, in the presence or absence of 100 mM DPC. The NMR experiments included HNCO, HNCA, HN(CA)CO, HNCACB, CBCA(CO)NH, CC(CO)NH, HCC(CO)NH and HBHA(CO)NH. The data were processed using nmrPipe with both forward and backward linear prediction for resolution improvement in each indirect dimension. Multi-dimensional NMR spectra were processed using nmrPipe and analyzed using nmrView for backbone resonance assignment.

### Secondary Structure Determination and Backbone Relaxation Analysis

The secondary structure of MPS-1(134–256) in the presence or absence of DPC micelles was determined using the TALOS+ program by inputting the chemical shift values of the backbone amide ^1^H, ^15^N, ^13^CO, ^13^C_α_, and ^13^C_β_ [20]. The relaxation parameter, ^15^N longitudinal relaxation T_1_, ^15^N transverse relaxation T_2_ and ^1^H-^15^N NOE, for backbone amide ^15^N nuclei, were acquired using a 600 MHz Bruker spectrometer at 25°C. Backbone ^15^N longitudinal relaxation T_1_ values were calculated from a series of ^1^H-^15^N correlation spectra with 11.2, 61.6, 142, 243, 364, 525, 757, and 1150 ms relaxation evolution delays. Backbone ^15^N transverse relaxation T_2_ values were obtained from the spectra with 0, 17.6, 35.2, 52.8, 70.4, 105.6, and 140.8 ms delays. Relaxation constants and the associated experimental errors were derived from single exponential curve-fitting of the peak heights using nmrView. Steady-state ^1^H-^15^N NOE intensities were obtained from the ratio *I*_NOE_/*I*_NO-NOE_, where *I*_NOE_ and *I*_NO-NOE_ are the peak heights in the NOE spectra with and without proton saturation, respectively.

## Results and Discussion

### Protein Expression and Purification

Previously, MPS-1 was predicted to contain a single transmembrane helix (46–68) [10], and further secondary structure prediction using PSIPRED verified the presence of a long stretch of hydrophobic residues that likely form a helix (see file S1). The truncated MPS-1(74–256) variant includes the entire cytosolic domain of MPS-1. Physiological studies of KVS-1 channel modulation by MPS-1 demonstrated the essential role of the MPS-1 cytosolic domain involves residues after residue Pro132 [13], suggesting our structural studies of the MPS-1(134–256) variant may provide insights of how the function of KVS-1 may be modulated by the serine/threonine kinase activity of MPS-1. As control, we also studied the full-length protein, which was purified from inclusion bodies in the presence of DPC micelles [21], [22]. In contrast, the truncated variants were purified in the soluble form in the presence or absence of DPC micelles. SDS-PAGE confirmed the successful purification of the proteins (Fig. 1C).

### Comparison of Solution NMR Spectra of MPS-1(134–256) and MPS-1(1–256) in DPC Micelles

**Figure 2:**
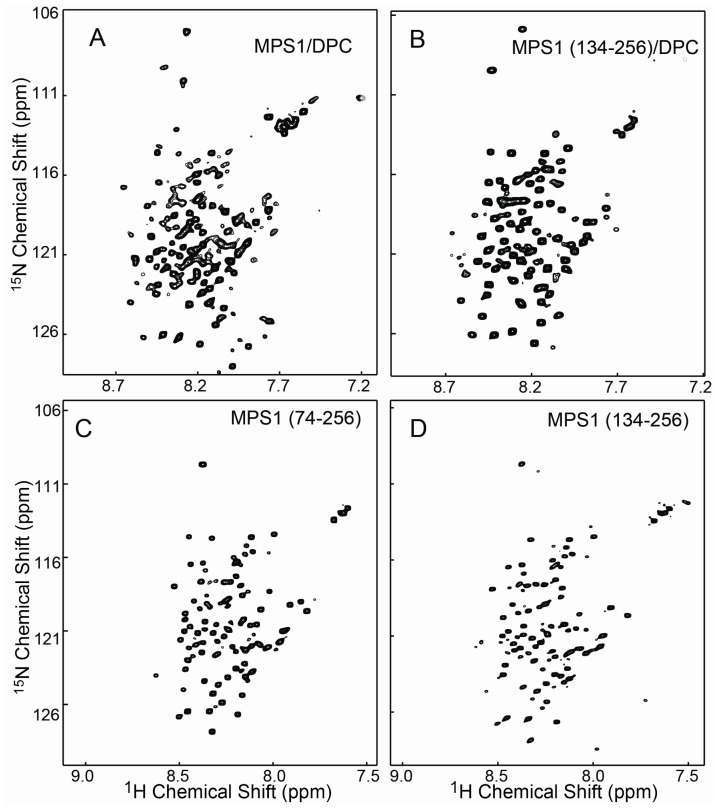
Two-dimensional ^1^H-^15^N HSQC spectra of ^15^N labeled full-length and truncated MPS-1 under different buffer conditions at 25°C. (A) Full-length MPS-1(1–256) spectrum in DPC micelles; (B) MPS-1(134–256) spectrum in DPC micelles; (C) MPS-1(74–256) spectrum in aqueous buffer; (D) MPS-1(134–256) spectrum in aqueous buffer.

In the 2D ^1^H-^15^N HSQC spectrum, each (cross peak) corresponds to a pair of ^1^H and ^15^N nuclei in the protein backbone amide groups. Since both ^1^H and ^15^N chemical shift values are very sensitive to local variations in protein conformation, the distribution of cross peaks in ^1^H-^15^N HSQC spectrum can be used to evaluate protein conformation under different conditions. In this study, 2D ^1^H-^15^N HSQC spectra were acquired for MPS-1(1–256), MPS-1(74–256) and MPS-1(134–256) in both the presence and absence of detergent micelles (Fig. 2).

Due to the presence of the hydrophobic transmembrane helix, detergent micelles were required to maintain the stable conformation of full-length MPS-1(1–256) in aqueous buffer. Only 132 peaks were discernable (Fig. 2 A) instead of ∼256. The unobserved peaks were possibly due to signal overlap or line-width broadening in the large micelles. The ^1^H-^15^N HSQC spectrum of MPS-1(134–256) showed 120 peaks (Fig. 2B), and most of the resonances overlapped with those of the full-length protein (Fig. S1A in file S1). The additional 12 cross speaks observed with the full-length MPS-1 presumably belonged to residues in other regions of the protein. The MPS-1(134–256) variant therefore shared a very similar tertiary structure in the cytosolic domain with the full-length protein, suggesting that the detergent micelles preserved the similar conformation of the full-length form.

### HSQC Spectra of MPS-1(74–256) and MPS-1(134–256) Exhibited High Similarity

The 2D ^1^H-^15^N HSQC spectra of MPS-1(74–256) and MPS-1(134–256) (Fig. 2C, D) showed similarly dispersed resonances suggesting that both variants adopted a comparable structure in aqueous solution in the absence of detergent. Overlaying the spectra revealed the presence of only a few extra peaks in the MPS-1(74–256) spectra (Fig. S1B in file S1), presumably belonging to residues between position 74–134.

### Backbone NMR Resonance Assignment of MPS-1(134–256) in Presence or Absence of DPC Micelles

**Figure 3:**
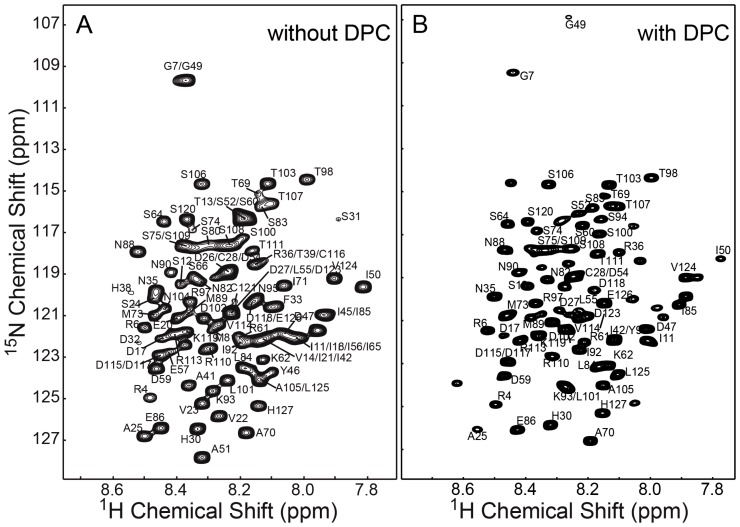
^1^H-^15^N HSQC spectra showing the crosspeak assignments of MPS-1(134–256) in the absence or presence of DPC micelles. (A) Identifies of a total of 90 of 111 non-proline amino acids were based on (N)H, N, C_α_, C_β_ and CO correlations were made for MPS-1(134–256); (B) In contrast, 66 amino acids were assigned for for MPS-1(134–256) in DPC micelles.

The HSQC spectra of MPS-1(134–256) in the presence (Fig. 2B) or absence (Fig. 2D) of DPC micelles showed clear differences in the distribution of cross peaks (Fig. S2 in file S1), possibly due to interactions between MPS-1(134–256) and the choline head groups of DPC micelles. In order to assign resonances for MPS-1(134–256) in the presence or absence of DPC micelles, two sets of triple resonance 3D NMR experiments were conducted with uniformly ^13^C- and ^15^N-enriched protein. A total of 90 backbone resonance assignments (NH, N, C_α_, C_β_ and CO) were achieved for MPS-1(134–256) in aqueous buffer for 111 non-proline residues (Fig. 3A), while 66 backbone resonance assignments were achieved in the presence of DPC micelles (Fig. 3B). The first ten residues could not be assigned in either case, indicating a high degree of flexibility in this region. The lower number of resonances assigned in the presence of micelles was most likely due to extensive line-width broadening of hydrophobic residues interacting with the micelles. Nevertheless, a similar resonance distribution profile was observed for MPS-1(134–256) in either aqueous or detergent-associated conditions.

### Secondary Structure and Backbone Relaxation Analysis of MPS-1(134–256)

**Figure 4:**
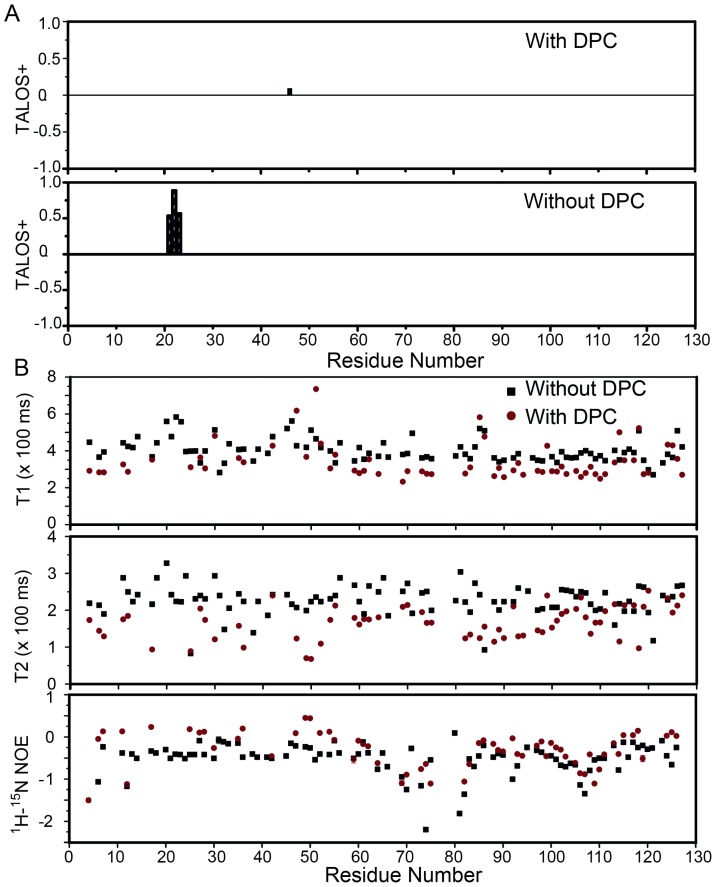
Secondary structure and backbone relaxation analysis of MPS-1(134–256) in the absence or presence of DPC micelles. (A) TALOS + secondary structure prediction were based on the assigned backbone ^13^CO, ^13^C_α_, ^13^C_β_ chemical shifts of MPS-1(134–256) in the presence or absence of DPC. Only random coil secondary structure was observed for MPS-1(134–256) in both aqueous buffer and DPC micelles. (See Fig. S3 in file S1 for the Y-axis scale from 0 to 0.3) (B) Distribution of the measured ^15^N T_1_ longitudinal and T_2_ transverse relaxation times and steady-state ^1^H-^15^N NOE values along the primary sequence in the absence (black) or presence (red) of DPC micelles.

Using the assigned backbone C_α_, C_β_ and CO chemical shift values, the program TALOS+ [20] was used to estimate the dihedral angles φ and φ of the residues within MPS-1(134–256) in the presence or absence of DPC micelles [20]. Normally, a positive TALOS+ index value indicates a β-sheet secondary structure, while a negative value indicates a α-helical secondary structure. Since the amplitude of the TALOS+ index is a measure of the validity of the predicted secondary structure, a value of zero indicates a random coil secondary structure. Surprisingly, most of the residues in MPS-1(134–256) had TALOS+ index close to zero (Fig. 4A), indicating a random coil secondary structure in both the presence and absence of DPC micelles. Three positive TALOS+ index values were observed for residues 154, 155 and 156 (the residue 21, 22 and 23 starting from residue 134) in the absence of micelles, indicating a β-sheet secondary structure in this region, which was consistent with the predicted β-extended structures around residues 154, 155 and 156 predicted using PRIPRED (see file S1).

To verify the secondary structure results, backbone relaxation analysis of MPS-1(134–256) was conducted in the presence or absence of DPC micelles. Backbone ^15^N T_1_ longitudinal relaxation time, T_2_ transverse relaxation time, and steady-state ^1^H-^15^N NOE values were measured for uniformly ^15^N-labeled protein (Fig. 4B). T_1_ longitudinal relaxation values were clustered around 300 or 400 ms for MPS-1(134–256) in both the presence or absence of DPC micelles (Fig. 4B, upper row), whereas T_2_ transverse relaxation values were approximately 150 or 250 ms (Fig. 4B, middle row). Steady-state ^1^H-^15^N NOE values were below zero for MPS-1(134–256), again in both conditions (Fig. 4B, lower row). For a stably-folded protein of a similar size (T4 lysozyme, 130 residues), T_1_ and T_2_ values were approximately 500 ms or 150 ms, respectively [23]. Normally, the rotational correlation time of a globular protein (τ_c_) shares a linear relationship with the T_1_/T_2_ ratio. The calculated T_1_/T_2_ ratio for MPS-1(134–256) in aqueous buffer (∼1.6) was around half that of T4 lysozyme (∼3.3), indicating that MPS-1(134–256) was not in a stably-folded conformation, but rather in a high flexibility unfolded state in aqueous buffer. The relatively large T_1_/T_2_ ratio of MPS-1(134–256) (∼ 2.0) may be due to the high viscosity in the presence of detergent micelles. Another very strong indication of protein flexibility was the backbone steady-state ^1^H-^15^N NOE values. A positive value (greater than 0.7) indicates a rigid conformation, while a negative value indicates high flexibility (mostly around −0.4). In contrast, many of the ^1^H-^15^N NOE values for T4 lysozyme were at least 0.8 [23].

### The Highly Flexible Random Coil Conformation suggests MPS-1 is not a Ser/Thr Kinase

The backbone T_1_ and T_2_ relaxation and heteronuclear NOE values measured in this study indicated a highly flexible random coil secondary structure for the truncated MPS-1(134–256). Furthermore, TALOS+ predicted a disordered structure along the entire sequence in both the presence and absence of DPC micelles. This raises the important question of how an intrinsically unstructured C-terminal region could possibly possess the previously reported serine/threonine kinase activity. Several conserved residues were considered to be indicative of kinase activity. Residues D179, F180 and G181 are conserved among kinases and are involved in chelating the Mg^2+^ ions associated with ATP. Residues H162, S163, and D164 are believed to be catalytic residues that may act as general acids and bases important for phosphoryl transfer. However, for all serine/threonine kinases characterized to date, the conserved residues around the ATP binding pocket reside in stable conformations and defined secondary structures [24]. The highly flexible random coil structure of the MPS-1(134–256) therefore is not consistent with this function. The random coil secondary structure prevented any further efforts to determine the 3D structure of MPS-1(134–256) in any more detail using conformational restraints.

All active serine/threonine kinase enzymes characterized to date have defined α-helical and β-sheet secondary structures that assemble into the tertiary and quaternary structures characteristic of the enzyme family. Protein kinase A, B, C, D, and G subfamilies all belong to the wider AGC family that are characterized by a C-terminal extension of the kinase domain that includes one or more regulatory phosphorylation sites that are important for kinase activity [14]. The AGC kinase was originally characterized by Steven Hanks and Tony Hunter in 1995, and defined the subgroup of Ser/Thr protein kinases most closely related to PKA, PKG and PKC based on sequence alignment of the catalytic kinase domain [14]. All AGC kinase domain structures determined to date exhibit the typical bilobal kinase fold first described for PKA [25]. Taken together, the random coil structure of the cytoplasmic domain of MPS-1 determined in this study strongly indicates that this protein is not an active serine/threonine kinase.

Despite the flexible random coil structure of MPS-1(134–256), there remains the possibility that MPS-1 adopts a functional secondary and tertiary structure upon forming a complex with the KVS-1 channel or other binding partner. Unfortunately, the large size of the tetrameric KVS-1 channel prevented us from investigating this potential interaction by solution NMR. MPS-1 shares high sequence similarity with other β-auxiliary proteins such as MiRP1 and MiRP3 that have been reported to associate with and modulate potassium channels [10]. Solution NMR and backbone relaxation studies of MiRP1 (KCNE2) illustrated a flexible C-terminal domain, and truncations of KCNE2 showed that this domain modulated the activity of KCNQ1 [26].

Protein domain flexibility can be important for protein-protein interactions, and intrinsically unstructured proteins can act as hubs upon which other proteins can bind. However, enzyme–substrate recognition and catalysis involve formation of a stable complex that requires some degree of rigidity [27]. Even so, the RS domain of the serine/arginine-rich splicing factor 1 was recently reported to perform a dramatic conformational switch from a fully disordered state to a partially rigidified arch-like structure upon phosphorylation [28]. Such a mechanism has yet to be reported for kinases, and would require the spontaneous formation of extensive α-helical regions. Further *in vivo* biophysical and functional studies of MPS-1 in *C. elegans* are necessary to confirm whether MPS-1 modulates KVS-1 channel current via serine/threonine kinase activity.

In summary, full-length and truncated MPS-1 were over-expressed in *E. coli* and purified in the presence or absence of DPC micelles. The similarity of the ^1^H-^15^N HSQC spectra of the full-length and MPS-1(134–256) truncated variant in DPC micelles indicated that the C-terminal cytosolic domain shares a similar structure in both proteins. Backbone resonance assignment of MPS-1(134–256) in the presence or absence of DPC micelles provided the basis for site-specific secondary structure and relaxation analysis. TALOS+ and backbone relaxation experiments clearly showed that the C-terminal cytosolic domain of MPS-1 (residues 134–256) adopted a highly flexible random coil structure. Such a structure would not be expected to support the reported serine/threonine kinase activity, which indicates that MPS-1 does not modulate KVS-1 channel function via a phosphorylation mechanism.

## Supporting Information

File S1 **Supporting information.** Figure S1, NMR spectra overlay of full-length and truncated MPS-1. Figure S2, NMR spectra overlay of MPS-1(134–256) in the absence and presence of DPC micelles. Figure S3, TALOS + secondary structure calculation prediction were based on the assigned backbone 13CO, 13Cα, 13Cβ chemical shifts of MPS-1(134–256) in the presence of DPC.
(DOC)
