# Supplementary material for: Solution NMR of MPS-1 Reveals a Random Coil Cytosolic Domain Structure
Source: PLoS One. 2014 Oct 27;9(10):e111035. doi: 10.1371/journal.pone.0111035 (PMC4210162; doi:10.1371/journal.pone.0111035)

**Solution NMR of MPS-1 reveals a random coil cytosolic domain structure**

Pan Li^1,#^, Pan Shi^2, #^, Chaohua Lai^1^, Juan Li^1^, Yuanyuan Zheng^1^, Ying Xiong^1^, Longhua Zhang^1,*^, Changlin Tian^1,2,*^

1. Hefei National Laboratory of Microscale Physical Sciences, School of Life Sciences, University of Science and Technology of China, Hefei, Anhui, P. R. China
2. High Magnetic Field Laboratory, Hefei institutes of Physical Science, Chinese Academy of Sciences, Hefei, Anhui, P. R. China

**Secondary Structure prediction using PSIPRED (**[**http://bioinf.cs.ucl.ac.uk/psipred/**](http://bioinf.cs.ucl.ac.uk/psipred/) **c: random coil; h: helix; e: extended sheet)**

**MHKNISPTCQEGIREACITLKNACDPQVLDKAAIRMREYENEFGRILYTI**

**cccccccchhhhhhhhhhhhhhcccccchhhhhhhhhhhhhhhhhhhhhh**

**|🡨74**

**SILIMFSFVIILLMVRSIRRTQSTVEMDSLLDAMRIREELEIQERKRRRL**

**hhhhhhhhhhhhhhhhhcccccchhhhhhhhhhhhhhhhhhhhhhhhhhh**

**|🡨134**

**MRAKTQVTAWLVNKNKEKGPEKRKDSEWKPLPNGTRPRGHYSISTVTSDI**

**hhhhhhhhhhhhhhccccccccccccccccccccccccccceeeeecccc**

**PEIVVSADDCIHSDFPNRPHTPAISMIYDFGIASPDLIEPDSRKPSISSS**

**cceeeccccccccccccccccceeeeeeeccccccccccccccccccccc**

**TAIPMSSSSSSSMNSLIEPNMNSIKSNPRTYSLDTNASTSSRTPRVDCDD**

**ccccccccccccccccccccccccccccceeeeccccccccccccccccc**

**KSCLDV**

**Cccccc**

**Transmembrane Helix Prediction using TMHMM program:**

**(http://www.cbs.dtu.dk/services/TMHMM-2.0/)**

**
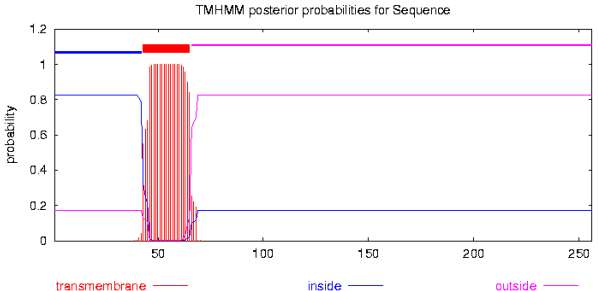
**

**Figure S1. NMR spectra overlay of full-length and truncated MPS-1.** (A) ^1^H-^15^N HSQC spectra of MPS-1(134-256) versus MPS-1(1-256) in DPC micelles. (B) ^1^H-^15^N HSQC spectra of MPS-1(74-256) versus MPS-1(134-256) in aqueous buffer.

**
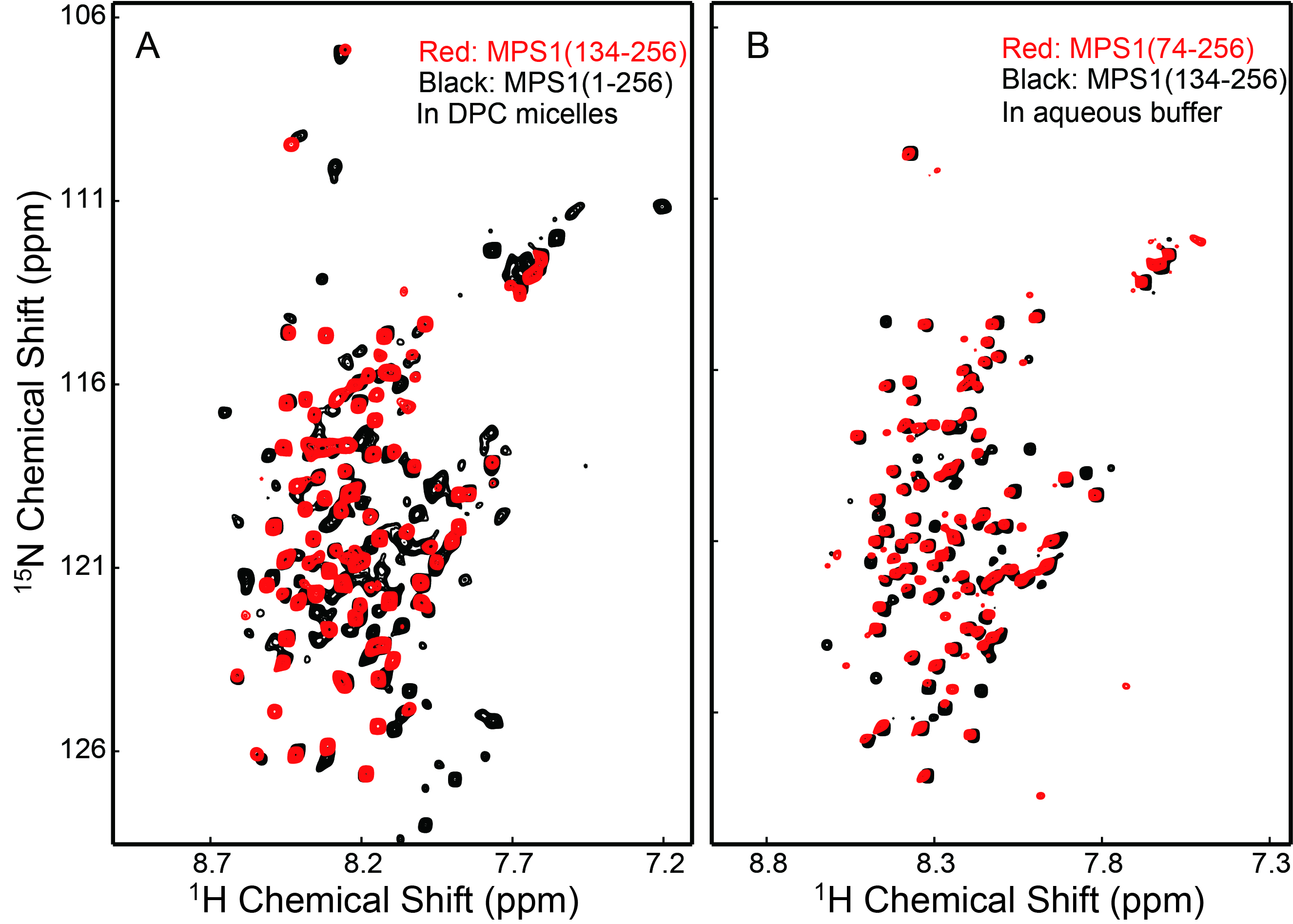
**

**Figure S2. NMR spectra overlay of MPS-1(134-256) in the absence and presence of DPC micelles.** Red: ^1^H-^15^N HSQC spectrum of MPS-1(134-256) in DPC micelles. Black: ^1^H-^15^N HSQC spectrum of MPS-1(134-256) in aqueous buffer.


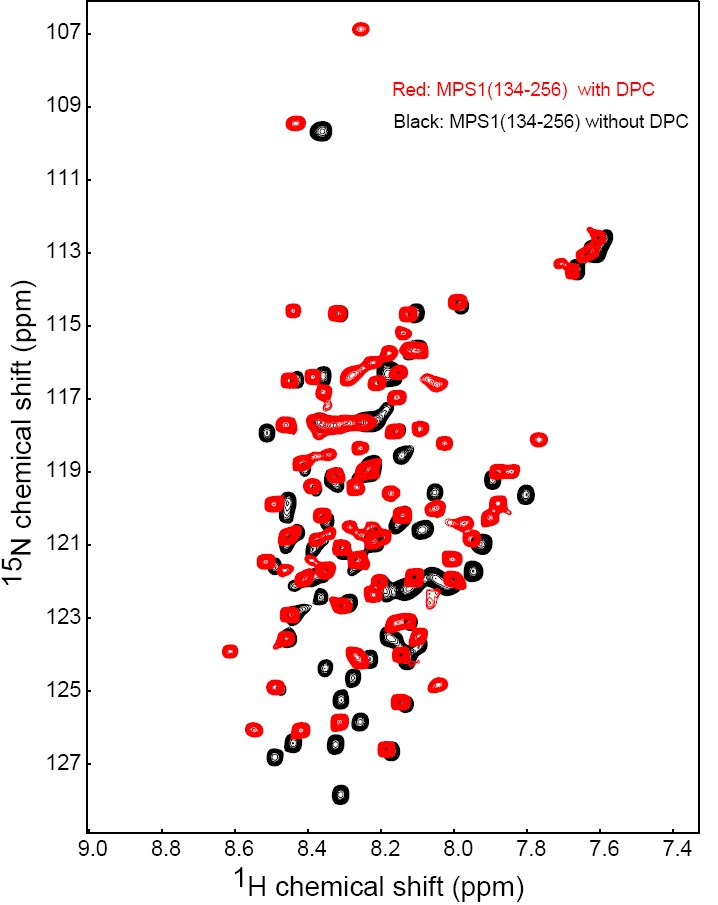


**Figure S3. TALOS + secondary structure calculation prediction were based on the assigned backbone ^13^CO, ^13^C_α_, ^13^C_β_ chemical shifts of MPS-1(134-256) in the presence of DPC.** Only random coil secondary structure was observed for MPS-1(134-256) in DPC micelles. The Y-axis scale in the TALOS+ prediction is from 0 to 0.3.


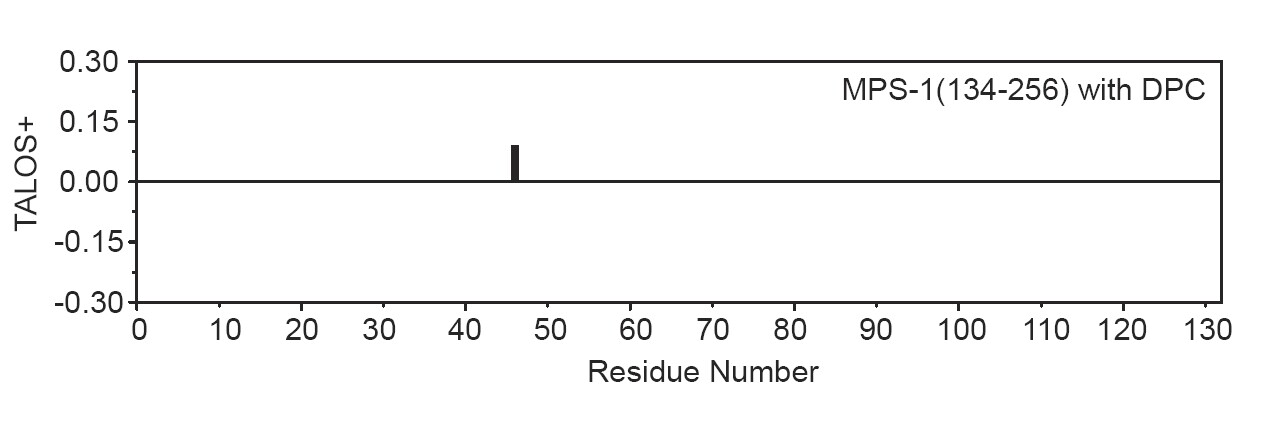

Supplement: File S1 — Supporting information. Figure S1, NMR spectra overlay of full-length and truncated MPS-1. Figure S2, NMR spectra overlay of MPS-1(134–256) in the absence and presence of DPC micelles. Figure S3, TALOS + secondary structure calculation prediction were based on the assigned backbone 13CO, 13Cα, 13Cβ chemical shifts of MPS-1(134–256) in the presence of DPC. (DOC) [file pone.0111035.s001.doc]
